# Protocol for the induction of human spinal motor neurons from human induced pluripotent stem cells for studying amyotrophic lateral sclerosis

**DOI:** 10.1016/j.xpro.2025.104016

**Published:** 2025-08-06

**Authors:** Selena Setsu, Satoru Morimoto, Shiho Nakamura, Fumiko Ozawa, Hideyuki Okano

**Affiliations:** 1 Keio University Regenerative Medicine Research Center, Kanagawa 210-0821, Japan; 2 Laboratory of RNA Function, Institute for Quantitative Biosciences, The University of Tokyo, Tokyo 113-0032, Japan; 3 Department of Computational Biology and Medical Sciences, Graduate School of Frontier Sciences, The University of Tokyo, Tokyo 277-0822, Japan; 4 Division of Neurodegenerative Disease Research, Tokyo Metropolitan Institute for Geriatrics and Gerontology, Tokyo 173-0015, Japan

**Keywords:** Cell Biology, Cell culture, Single Cell, Cell-based Assays, Microscopy, Molecular Biology, Gene Expression, Antibody, Neuroscience, Stem Cells, Cell Differentiation

## Abstract

Here, we present a protocol for inducing spinal lower motor neurons (LMNs) from human induced pluripotent stem cells (iPSCs). We describe steps for preparation of a chemically induced transitional state (CTraS), transduction with Sendai virus, and LMN differentiation and maintenance. We then detail procedures for live imaging for single-cell-based survival analysis and neurite length of LMNs using BioStation and immunocytochemistry for induction efficiency check. This protocol is optimized for amyotrophic lateral sclerosis (ALS) research and large-scale screening.

For complete details on the use and execution of this protocol, please refer to Setsu et al.^1^

## Highlights

• Step-by-step procedure for inducing motor neurons
• Live imaging procedure for tracking cell survival and neurite extension
• Immunocytochemistry procedure for measuring induction efficiency

## Abstract (editor highlights)

Publisher’s note: Undertaking any experimental protocol requires adherence to local institutional guidelines for laboratory safety and ethics.

## Graphical abstract

**Figure:**
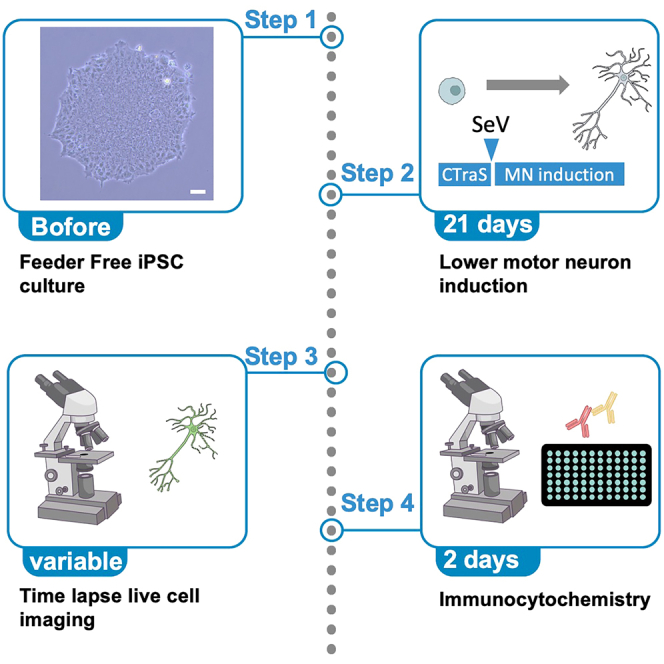

## Before you begin

### Innovation

This protocol presents a rapid and efficient method for inducing human spinal lower motor neurons (LMNs) from iPSCs, significantly advancing existing ALS modeling and drug screening workflows. By combining a chemical transitional embryoid body-like state (CTraS) pretreatment with Sendai virus (SeV) -mediated transcription factor induction, we achieved LMN induction efficiencies of 70–80% within two weeks—substantially faster and more efficient than conventional methods, which often require longer durations and achieve only ∼30% efficiency. The CTraS method uses small molecules (SB431542, dorsomorphin, CHIR99021) to inhibit TGF-β/BMP and activate Wnt signaling, enhancing responsiveness to transcription factors. Our protocol also integrates single-cell-resolution evaluation of neuronal morphology and survival using time-lapse imaging, enabling high-content phenotypic screening. The speed, scalability, and endpoint clarity of our system make it well-suited for large-scale screening of iPSC lines from sporadic ALS patients—overcoming previous limitations associated with disease heterogeneity and low throughput. Furthermore, our approach lays the groundwork for precision medicine, as it can accommodate personalized screening timelines crucial for rapidly progressing diseases like ALS. This protocol thus provides an integrated and scalable platform that combines high induction efficiency with single-cell survival analysis, enhancing both mechanistic ALS research and drug discovery efforts.

### Institutional permissions

This study was conducted in accordance with the Declaration of Helsinki and was approved by the ethics committee of Keio University School of Medicine (Approval no. 20080016).

This protocol involves the use of SeV. Conducting SeV experiments requires a dedicated room and biosafety cabinet that meet Biosafety Level 2 (BSL-2) standards, along with institutional approval for BSL-2 work.

### iPSC preparation

**Timing:About 5 days before**

This section describes how to culture and prepare human induced pluripotent stem cells (iPSCs) for motor neuron induction. Proper iPSC maintenance and passaging are critical for ensuring cell health and reproducibility of differentiation.

1. Culture human iPSCs in feeder-free conditions using StemFit AK02N medium. The iPSC lines used in this study (201B7 and A3411) are available from the RIKEN BioResource Research Center.
2. iPSC passage.
  a. Aspirate medium gently and wash with sterile 1× PBS (−).
  b. Aspirate the liquid and gently add the sterile 1× PBS (−).
  c. Aspirate the PBS (−) and disassociate the iPSC with fresh medium containing 10 μM Y27632 and iMatrix-511 by pipetting.
  d. Count the cells.
  e. Seed 0.3 × 10^4^–1 × 10^4^ cells in 6 well plate.

***Note:*** The seeding cell number differs cell line to cell line. You need to adjust the cell number so that it will not be too confluent after a chemically transitional embryoid-body-like state (CTraS) preparation.^2^

**CRITICAL:** Ensure uniform cell density to achieve consistent induction efficiency. Im7mediately after seeding, gently rock the plate in a cross pattern to evenly distribute the cells across the surface.

3. Maintain cells at 37°C in 5% CO_2._ Change medium at the next day of passage. After that, change the medium every day or every other day.

**CRITICAL:** Use freshly passaged cells at 70%–80% confluence for optimal differentiation. Prior to differentiation, ensure the cells are passaged at least once after thawing from cryopreservation.

## Key resources table

**Table:** 

REAGENT or RESOURCE	SOURCE	IDENTIFIER
**Antibodies**		

MNR2/HB9/Mnx1 monoclonal antibody, dilution 1:150	DSHB	81.5C10-C
Anti-isl1, dilution 1:1,000	DSHB	39.4D5-C
Purified anti-Tubulin β 3 (TUBB3) antibody, dilution 1:2,000	BioLegend	801202
Goat anti-mouse IgG1 cross-adsorbed secondary antibody, Alexa Fluor 488, dilution 1:2,000	Invitrogen	A21121
Goat anti-mouse IgG2b cross-adsorbed secondary antibody, Alexa Fluor 488, dilution 1:2,000	Invitrogen	A21141
Goat anti-mouse IgG2a cross-adsorbed secondary antibody, Alexa Fluor 647, dilution 1:2,000	Invitrogen	A21241
SeV-Lhx3-Ngn2-Isl1	ID Pharma	N/A
SeV-Lhx3-Ngn2-Isl1-EGFP	ID Pharma	N/A
SeV-LHX3-NGN2-ISL1	Repli-tech	N/A
SeV-LHX3-NGN2-ISL1-mEmerald	Repli-tech	N/A

**Chemicals, peptides, and recombinant proteins**		

StemFit AK02N medium	Ajinomoto	AK02N
SB431542	Sigma-Aldrich	S4317
CHIR99021	Cayman Chemical	13122
Dorsomorphin	Santa Cruz	sc-202597
Matrigel	Thermo Fisher Scientific	354277
iMatrix-511-silk	Matrixome	892 021
PLL	Sigma-Aldrich	N/A
KBM neural stem cell medium	KOHJIN BIO	16050400
B27 supplement	Thermo Fisher Scientific	17504-001
DAPT	Sigma-Aldrich	D5942-5MG
Penicillin-streptomycin	Thermo Fisher Scientific	15140-122
Ascorbic acid (AA)	Sigma-Aldrich	A4544
Brain-derived neurotrophic factor (BDNF)	R&D Systems	248-BD
Glial cell-derived neurotrophic factor (GDNF)	Alomone Labs	G240
Retinoic acid	Sigma-Aldrich	R2625
PD0332991	Sigma-Aldrich	PZ0199
TrypLE Select	Thermo Fisher Scientific	12563-029
Y27632	Nacalai	18188-04
Paraformaldehyde 16%	Electron Microscopy Sciences	15710
Goat serum	MBL	EQ-102
Triton X-100	Nacalai	31233-42
NaN_3_	Nacalai	31233-42

**Experimental models: Cell lines**		

Human: hiPSC, 201B7	Takahashi et al.^3^	HPS0063: CVCL_A324
Human: hiPSC, *TARDBP* M337V, A3411	Egawa et al.^4^	HPS0292: CVCL_T781

**Software and algorithms**		

CL-Quant	Nikon	N/A
CL-Quant: Single-cell tracking algorithm	Nikon	Custom order
CL-Quant: Neurite length algorithm	Nikon	Custom order

**Other**		

BioStation CT	Nikon	N/A
IN Cell Analyzer 6000	Cytiva	N/A

## Materials and equipment

**Table:** Chemical induction medium

Reagent	Final concentration	Amount
StemFit AK02N medium	N/A	10 mL
SB431542 (10 mM)	3 μM	3 μL
CHIR99021 (10 mM)	3 μM	3 μL
Dorsomorphin (5 mM)	3 μM	6 μL
**Total**	**N/A**	**10 mL**

Use freshly prepared medium.

**Table:** Coating solution

Reagent	Final concentration	Amount
PBS (−)	N/A	20 mL
Matrigel	10 μL/mL	200 μL
iMatrix-511 silk	6.4 μL/mL	128 μL
**Total**	**N/A**	**20.328 mL**

Thaw the Matrigel gradually by pipetting and mixing with cold PBS (−) (4℃) and then add the iMatrix-511 silk. The PBS (−) and Matrigel solution can be stored at 4°C up to one week. Add iMatrix-511 silk before use.

**Table:** KBM medium

Reagent	Final concentration	Amount
KBM Neural Stem Cell	N/A	500 mL
B27	2%	10 mL
Penicillin-streptomycin	100 U/mL / 100 μg/mL	5 mL
**Total**	**N/A**	**515 mL**

Store at 4°C for 2 weeks.

**Table:** MN medium

Reagent	Final concentration	Amount
KBM medium	N/A	10 mL
Ascorbic acid (200 mM)	200 μM	10 μL
BDNF (10 μg/mL)	10 ng/mL	10 μL
GDNF (10 μg/mL)	10 ng/mL	10 μL
RA (20 mM)	1 μM	0.5 μL
**Total**	**N/A**	**10 mL**

Store at 4°C for 2–3 days.

**Table:** Blocking buffer

Reagent	Final concentration	Amount
PBS (−)	N/A	9 mL
Goat serum	10%	1 mL
20% Triton X-100	0.30%	150 μL
**Total**	**N/A**	**10 mL**

Store at 4°C for 2 weeks.

## Step-by-step method details

### Preparation of chemically induced transitional state—Days 1–7

**Timing: 7 days**

A chemically transitional embryoid body-like state (CTraS) can be induced through a simple treatment with three small molecules—SB431542, Dorsomorphin, and CHIR99021. This treatment enhances the differentiation potential of human pluripotent stem cells (hPSCs) into all three germ layers.

**Figure 1:**
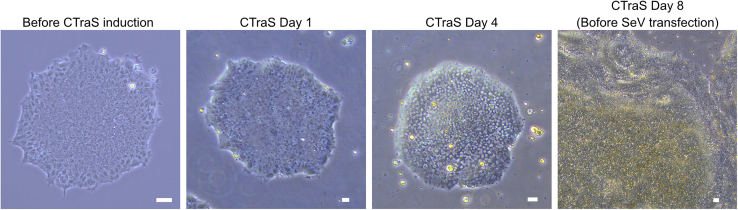
Progression of CTraS induction As induction progresses, each colony expands, and the center of the colony becomes elevated. Scale bar: 100 μm.

1. Replace iPSC medium with 2 mL chemical induction medium.
2. Change medium daily.
3. Observe cells under a microscope to ensure the formation of an embryoid body-like state (Figure 1).

***Note:*** If you succeed at forming CTraS cells, the middle of each colony rises as in the Figure 1, CTraS day 4 and 8.

### Transduction with Sendai virus—Days 7 and 8

**Timing: 2 days**

This section outlines the viral transduction of iPSCs with transcription factors (Lhx3, Ngn2, and Isl1) using SeV vectors. This step initiates directed differentiation into spinal lower motor neurons.

**CRITICAL:** All the procedures that involve SeV handling must be done in a room and a safety cabinet that satisfy the Biosafety level 2 (BSL-2) requirements and have approval for BSL-2 experiment.

4. PLL coating.
  a. Put 0.0001% PLL (diluted with distilled water) to that the bottom of the well is covered.
  b. Place the plates in a 37°C incubator over 12 h.
5. Matrigel and iMatrix-511 coating.
  a. After aspirating the 0.0001% PLL, cover the well bottom with coating solution. Place the plates in a 37°C incubator at least 1 h before use.
6. Prepare MN medium supplemented with 10 μM Y27632, 10 μM DAPT and SeV (MOI 5).

***Note:*** The SeV composition and cell seeding density differ according to the purpose of the experiment. Please refer to Table 1 for the details.

***Note:*** For example, if the titer of SeV is 1.0 × 10^9^ CIU/mL and you seed 1 × 10^6^ cells in 500 μL medium per well, you add 10 μL SeV per 1 mL medium.

***Note:*** The transgenes introduced via SeV can consist of either human transcription factor genes (LHX3, NGN2, ISL1) or mouse orthologs (Lhx3, Ngn2, Isl1). The SeVs are commercially obtained through custom order. For further details, please refer to the key resources table section.

**CRITICAL:** Always Keep the SeV on ice during handling to prevent inactivation. Return the virus stock to a −80°C freezer immediately after preparing the medium. Avoid repeated freeze–thaw cycles; for optimal transduction efficiency, the virus should not be thawed more than twice.

7. Dissociate iPSC colonies into single cells using 0.5× TrypLE Select.
  a. Aspirate medium gently and wash with sterile 1 mL 1× PBS (−).
  b. Add 0.5× TrypLE Select (TrypLE Select:PBS(−) =1:1) just to cover the cells and incubate in 37°C for 5 min. 350 μL if you use 6 well plate
  c. Aspirate the liquid and gently add the sterile 1 mL 1× PBS (−).
  d. Aspirate the PBS (−), then disassociate the iPSC with 1 mL StemFit AK02N medium and vigorously pipetting to spray the medium across the well bottom.

***Note:*** Try not to make bubbles for accurate cell counting.

8. Count the cells.

***Note:*** As cell density is high, we separate 10 μL of cell suspension and dilute it to 1/10 for cell counting.

9. Collect desired number of cells and infect with SeV.
  a. According to cell count, calculate the amount of cell suspension you need.
  b. Dispense the cell suspension into a 1.5- or 15-mL tube.
  c. Centrifuge at 300 × *g* for 5 min. Bring out the seeding well plates from the incubator.
    ***Note:*** If the amount of cell suspension is small (<15 μL) you can skip the centrifugation and medium removal. Add the MN medium directly (step e).
  d. Carefully remove the medium without touching the cell pellet. There is no need to remove the medium completely.
  e. Add MN medium you prepared at step 3. Pipette gently and resuspend the cell.
    ***Note:*** If the volume of MN medium to be added exceeds 500 μL, first resuspend the cells in 250–500 μL of medium to ensure a single-cell suspension, then add the remaining volume.
  f. Aspirate the coating medium from a well and the cell suspension into the well. Gently shake the plate so that the cells spread evenly in the well.
  g. Place the plates in a 37°C, 5% CO_2_, 4% O_2_ incubator.
    ***Optional:*** 4% O_2_ setting is optional. It may result in better differentiation efficiency.^5^
10. Observe the cells under a microscopy at 24-, 48- and 72-h post-transduction morphological changes such as neurite extension, indicating successful transduction. If a SeV encoding EGFP along with transcription factors is used, assess fluorescence as well. Fluorescence intensity is typically sufficient to initiate downstream experiments by 72 h after transduction (Figure 3).

**Table 1:** Seeding density when transfecting SeVs

Aim	Plate size	SeV	Cell density	Medium amount at the day of seeding	Medium change amount
ICC	96-well glass bottom plate	SeV-Lhx3-Ngn2-Isl1	1 × 10^5^ cells/well	100 μL	200 μL
BioStation neurite length	12-well plate	SeV-Lhx3-Ngn2Isl1-EGFP	1 × 10^6^ cells/well	500 μL	1 mL
BioStation single cell tracking	12-well plate	SeV-Lhx3-Ngn2-Isl1 and SeV-Lhx3-Ngn2-Isl1EGFP (10:1 mixture)	6 × 10^5^ cells/well	500 μL	1 mL

### LMN differentiation and maintenance—Days 9–21

**Timing: 7–13 days**

This section describes the culture conditions required to support the differentiation and survival of induced lower motor neurons (LMNs) following transduction. Proper medium exchange and environmental conditions are essential for maintaining LMN identity and viability.

11. On the day following transduction, replace the medium with Day 9 MN medium supplemented with 10 μM DAPT (1 mL per well for a 12-well plate and 200 μL per well for a 96-well plate) (see Table 2).
12. Change the medium every 2–3 days. The schedule for full or half medium changes, along with the corresponding supplements to be added, is detailed in Table 2.
13. If the purpose is to check the differentiation efficiency, proceed to Immunocytochemistry at day 12.

**Figure 2:**
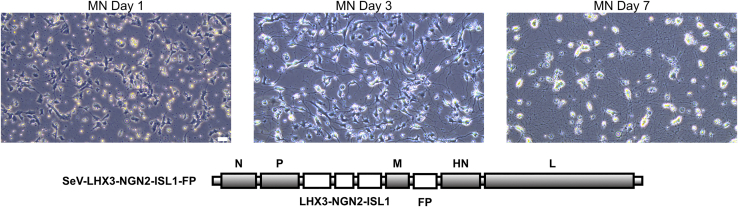
Progression of MN induction Cell morphology changes markedly during the first week. By day 7, cells exhibit typical neuronal characteristics, including extended neurites. Scale bar: 100 μm. An example of SeV vector construction is illustrated.

**Figure 3:**
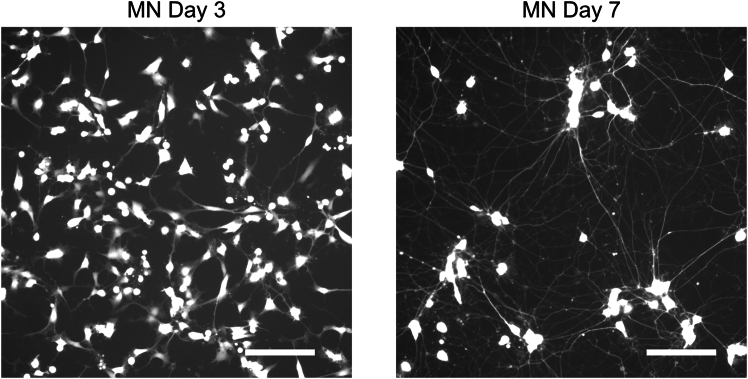
GFP expression 3 and 7 days after SeV transduction Scale bar: 160 μm. FP: fluorescent protein. Figures adopted from Setsu et al.^1^ with permission from Elsevier.

***Note:*** Cell morphology changes markedly during the first week, and by day 7, the cells exhibit typical neuronal characteristics with extended neurites and GFP expression (Figures 2 and 3).

***Note:*** Do not use an aspirator when removing the medium, as it may damage the cells and detach neurites. Instead, carefully remove the medium with a pipette and gently add fresh medium along the well wall.

**Table 2:** Medium change schedule

Day	Day 9	Day 11 (only for BioStation)	Day 12	Day 15	Day 18	Day 21
Change amount	full	full	full	half	full	half
Medium	MN medium	MN medium	MN medium	2× concentration MN medium	MN medium	2× concentration MN medium
Supplement	10 μM DAPT	10 μM DAPT	10 μM DAPT, 2 μM PD	20 μM DAPT, 4 μM PD

### Live imaging for single-cell-based survival analysis of LMNs using BioStation—Days 11–21

**Timing: Variable**

This section explains how to perform long-term live imaging using BioStation for tracking the survival of individual LMNs. The imaging data generated here are used to evaluate cellular vulnerability and disease phenotypes in ALS models at single-cell resolution.

14. Place 12-well plates with differentiating LMNs onto BioStation imaging platform, calibrate the focus and stage to ensure cell stability.

***Note:*** Pre-condition BioStation CT (Nikon) to maintain a stable environment (37°C, 5% CO_2_, and controlled humidity). Exposure times and magnifications are provided in Table 3.

15. Set up time-lapse imaging of 5 × 5 tiling images with an interval of 6 h. Use a ×10 objective lens for phase contrast and GFP images to track cell behavior dynamically.

**CRITICAL:** Maintain constant fluorescence intensity settings across imaging sessions to allow quantitative analysis. Representative time-lapse recordings used for survival and neurite analyses are provided as Methods videos S1, S2, S3, and S4.

**CRITICAL:** Avoid using high exposure intensity or prolonged exposure time to prevent phototoxicity, which can diminish EGFP fluorescence.

16. Use CL-Quant software for image processing, including background subtraction and tracking of dynamic processes.
17. Export high-quality image sequences and corresponding data for further analysis in software such as ImageJ or GraphPad Prism.

***Note:*** We recommend initiating tracking and survival analysis 30 h after mounting the imaging plate onto the BioStation when developing your own algorithm.

**Table 3:** BioStation setting

	Channel	Magnification	Exposure time (ms)	Luminance
Single cell tracking	Ph	10	4	60
	GFP	10	300	200
Neurite length	Ph	10	4	60
	GFP	10	350	200

Methods video S1. Phase-contrast time-lapse imaging for survival analysis, related to step 15

Methods video S2. GFP time-lapse imaging for survival analysis, related to step 15

Methods video S3. Phase-contrast time-lapse imaging for neurite analysis, related to step 19

Methods video S4. GFP time-lapse imaging for neurite analysis, related to step 19

### Live imaging analysis for neurite length of LMNs using BioStation—Days 11–21

**Timing: Variable**

This section provides the procedure for analyzing neurite outgrowth and morphology using time-lapse imaging. Measuring neurite length allows quantification of neuronal health and helps detect phenotypic differences in iPSC-derived LMNs under disease or treatment conditions.

18. Mount 12 well plates with differentiating LMNs onto BioStation, calibrate the focus and stage to ensure cell stability.

***Note:*** Pre-condition BioStation CT (Nikon) to maintain a stable environment (37°C, 5% CO_2_, and controlled humidity)

19. Set up time-lapse imaging of six image points per well with an interval of 12 h. Use a ×10 objective lens for phase contrast and GFP images.

**CRITICAL:** Maintain constant fluorescence intensity settings across imaging sessions to minimize phototoxicity and allow quantitative analysis.

20. Use CL-Quant software for image processing, including background subtraction and tracking of dynamic processes like neurite outgrowth. Quantify key metrics such as neurite length and cell survival duration using appropriate protocols—for example, in our case, a custom-ordered single-cell tracking algorithm for CL-Quant.
21. Export high-quality image sequences and corresponding data for further analysis in software such as ImageJ or GraphPad Prism.

### Immunocytochemistry for induction efficiency check

**Timing: 2 days**

This section describes the immunostaining procedure used to evaluate the efficiency of motor neuron induction. The staining targets LMN markers ISL1 and HB9 to quantify the proportion of successfully differentiated neurons and validate the overall quality of the protocol. Details of antibody dilutions are provided in Table 4.

22. Fix the cells.
  a. Confirm under the microscope that LMN cells at day 12 are healthy and not detached before fixation.
  b. Remove 150 μL medium from each well using an 8-channel pipette.
  c. Add 50 μL of 8% PFA (prepared by diluting 16% PFA in PBS) gently to the side wall of each well using single pipette.
  d. Incubate at 20°C–25°C for 20 min.
  e. Remove PFA with an 8-channel pipette.
  f. Add 50 μL of 4% PFA (prepared by diluting 16% PFA in PBS) gently to the side wall of each well using single pipette.
  g. Incubate at 20°C–25°C for 15 min.
  h. Wash cells twice with 100 μL of PBS.
  i. Add 100 μL of PBS per well. For storage, add 0.05% NaN_3_ in distilled water per well.

**Pause point:** You can store the plate at 4°C up to one week before proceeding to next step.

**CRITICAL:** Do not let the wells dry. Avoid pipetting directly onto the cells and always add through side wall.

23. Block non-specific binding.
  a. Remove liquids from each well.
  b. Add 50 μL of blocking buffer per well.
  c. Incubate for 1 h at 20°C–25°C.
24. Incubate with primary antibody.
  a. Prepare primary antibody dilution in PBS (−) + 5% FBS.
  b. Add 50 μL of diluted primary antibody per well.
  c. Incubate 16–20 h at 4°C, covered with aluminum foil.

***Note:*** Use appropriate antibody concentrations and validate with isotype controls if necessary.

25. Wash and apply secondary antibody.
  a. Prepare secondary antibody and Hoechst mix in same buffer as primary (PBS (−) + 5% FBS).
  b. Wash cells 3 times with 100 μL of PBS (−).
  c. Add 50 μL of secondary antibody solution per well.
  d. Incubate at 20°C–25°C for 30 min, covered with aluminum foil.
  e. Wash cells again 3 times with 100 μL PBS.
  f. Add 100 μL of 0.05% NaN_3_ per well and store at 4°C.
26. Acquire images using IN Cell Analyzer 6000 or other microscopes.

**Table 4:** Antibody and Hoechst dilution list

Primary antibody	Dilution	Secondary antibody	Dilution
**For HB9**			

MNR2/HB9/Mnx1 Monoclonal antibody	1:150	Goat anti-Mouse IgG1 Cross-Adsorbed Secondary Antibody, Alexa Fluor 488	1:2000
Purified anti-Tubulin β 3 (TUBB3) Antibody	1:2000	Goat anti-Mouse IgG2a Cross-Adsorbed Secondary Antibody, Alexa Fluor 647	1:2000
		Hoechst	1:500

**For Isl1**			

Anti-isl1	1:1000	Goat anti-Mouse IgG2b Cross-Adsorbed Secondary Antibody, Alexa Fluor 488	1:2000
Purified anti-Tubulin β 3 (TUBB3) Antibody	1:2000	Goat anti-Mouse IgG2a Cross-Adsorbed Secondary Antibody, Alexa Fluor 647	1:2000
		Hoechst	1:500

## Expected outcomes

**Figure 4:**
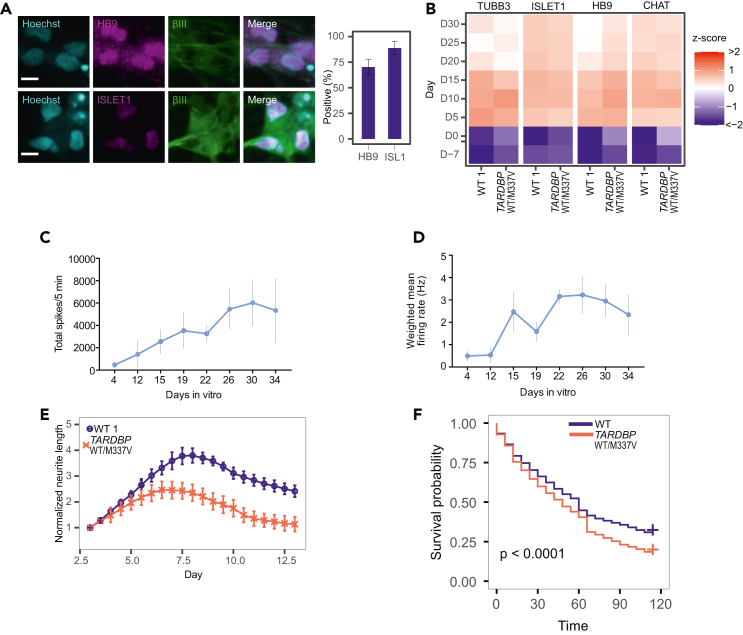
Functional, morphological, and survival phenotypes of induced lower motor neurons derived from iPSCs (A) Left: Representative immunocytochemistry image of induced lower motor neurons (LMNs) at day 7, stained for HB9 (magenta), ISLET1 (magenta), and TUBB3 (green), with Hoechst nuclear counterstaining (cyan). Scale bar = 10 μm. Right: Differentiation efficiency at day 7, quantified based on HB9 and ISLET1 immunostaining. Error bars represent standard deviation; n = 8 biological replicates (different iPSC lines). (B) Gene expression levels normalized by z-score; n = 3 technical replicates. (C) Total spike count, reflecting local field potential activity, measured over a 5-min MEA recording. Data represent mean ± SEM from 3 independent wells. (D) Weighted mean firing rate, calculated as the average firing rate adjusted for the number of active electrodes. Data represent mean ± SEM from 3 independent wells. (E) Total neurite length per well, normalized to the corresponding neurite length at day 3. (F) Survival analysis comparing ALS and wild-type control cell lines. P-values were determined using the log-rank test with Benjamini–Hochberg correction. All panels are reproduced or adopted from Setsu et al.^1^ with permission from Elsevier.

Researchers can achieve an induction efficiency of ∼80% for LMNs, characterized by *HB9* and *ISL1* expression, within two weeks (Figures 4A and 4B). LMNs generated using this protocol can be maintained in culture for at least four weeks post-differentiation (up to day 30), exhibiting stable neuronal morphology and sustained expression of motor neuron markers (Figure 4B). Functional assessments, such as spontaneous neuronal firing measured via MEA (Figures 4C and 4D), validate the maturity and activity of the derived LMNs. Live cell imaging using BioStation enables dynamic analysis of neurite outgrowth and cell survival duration in real time (Figures 4E and 4F), providing comprehensive insights into neuronal development and function. When using human iPSC line 201B7^4^ as healthy control and A3411 as TDP-43 M337V mutated ALS model,^5^ A3411 have shorter survival and maximal neurite length than 201B7 as presented in Figures 4E and 4F.

## Limitations

This protocol may result in variable efficiencies across different iPSC lines due to differences in genetic background. It is specifically optimized for the differentiation of lower motor neurons (LMNs) and may not be directly applicable to other neuronal subtypes. Live-cell imaging requires access to specialized equipment such as the BioStation, as well as expertise in image analysis. However, once time-lapse imaging is successfully acquired, standard tools such as ImageJ can be readily applied for cell tracking and neurite length quantification.

## Troubleshooting

### Problem 1

Differentiation efficiency is below 70% (steps 10 or 26).

### Potential solution

Confirm the confluence of iPSCs and optimize SeV infection conditions. Additionally, assess the success of CTraS treatment by immunostaining for germ layer–specific markers such as PAX6, BRACHYURY, NESTIN, and SOX17. If these markers are not detected, consider evaluating the quality or grade of the small molecules—SB431542, dorsomorphin, and CHIR99021—and adjust the cell passage number prior to CTraS induction to avoid excessive confluence after treatment.

### Problem 2

Cell death during transduction (steps 4–10).

### Potential solution

Ensure proper dissociation and minimize cell stress by optimizing plating density and medium conditions.

Check that cells are being handled properly during the following dissociation procedure:

Incubate the cells with 0.5× TrypLE Select for approximately 5 min at 37°C. Gently rock the well midway through the incubation to prevent dry spots from forming in the center. Avoid creating bubbles while pipetting to detach the cells, as this can cause excessive mechanical stress. Accurately count the cells to ensure correct seeding density as specified in Table 1. Include 10 μM Y-27632 (ROCK inhibitor) during and after dissociation to promote cell survival, particularly within the first 24 h after plating. Additionally, do not leave dissociated cells in the well for extended periods before seeding—ideally, proceed within 20 min.

### Problem 3

Image drift during live imaging (step 14).

### Potential solution

Calibrate the BioStation platform to minimize mechanical drift and stabilize the imaging chamber. Please also avoid changing the medium just before the scheduled imaging time, as it may cause condensation on the culture plate and lead to inaccurate autofocusing.

### Problem 4

Photobleaching during live imaging (step 14).

### Potential solution

Reduce light intensity and exposure time while ensuring adequate image quality.

### Problem 5

Neurite recognition algorithm does not recognize neurite properly (steps 16 and 20).

### Potential solution

Increase exposure time or adjust segmentation threshold so that the algorithm can distinguish background and neurite properly.

## Resource availability

### Lead contact

Requests for further information and resources should be directed to and will be fulfilled by the lead contact, Hideyuki Okano (hidokano@keio.jp).

### Technical contact

Technical questions on executing this protocol should be directed to and will be answered by the technical contact, Satoru Morimoto (satoru_morimoto@keio.jp).

### Materials availability

Materials used in this study are available upon request from the technical contact.

### Data and code availability

Data supporting the findings of this protocol are available upon reasonable request.

## Acknowledgments

This protocol was supported by grants from the Japan Society for the Promotion of Science (JSPS) and the Japan Agency for Medical Research and Development (AMED). We would also like to thank Shinya Yamanaka for kindly providing 201B7 and Haruhisa Inoue (Kyoto University) for kindly providing A3411. S.M. reports grant support from the 10.13039/501100001691Japan Society for the Promotion of Science (JSPS) (KAKENHI grant nos. JP21H05278, JP22K15736, 22K07500, and 25H00007), AMED (grant nos. JP23bm1123046 and JP23kk0305024), the 10.13039/501100005927Daiichi Sankyo Foundation of Life Science, the UBE Academic Foundation, the 10.13039/501100004051Kato Memorial Trust for Nambyo Research, the 10.13039/100020050Japan Intractable Diseases (Nambyo) Research Foundation (2024A04), and the 10.13039/501100003844Inamori Foundation during the conduction of the study. H.O. has grant support from 10.13039/501100001691JSPS (KAKENHI grant nos. JP20H00485, JP21F21410, JP21H05273, JP22KF0333, and JP25H00007) and 10.13039/100009619AMED (grant nos. JP20ek0109395, JP20ek0109329, JP21wm0425009, JP22bm0804003, JP23bm1423002, and JP25ek0109811). The funding sources had no role in the analysis.

## Author contributions

S.M. designed the protocol for cell culture. F.O., S.N., S.S., and S.M. performed the experiments. S.M. and S.S. analyzed the data and wrote the manuscript. S.M. and H.O. provided a grant for the study. H.O. corrected the manuscript and oversaw the research program.

## Declaration of interests

H.O. reports grants and personal fees from K Pharma, Inc., and SanBio Co., Ltd., outside the submitted work.

## Declaration of generative AI and AI-assisted technologies in the writing process

During the preparation of this work, the authors used ChatGPT by OpenAI in order to improve the clarity, grammar, and professional tone of the manuscript. After using this tool, the authors reviewed and edited the content as needed and take full responsibility for the content of the publication.
